# Multisensory decisions from self to world

**DOI:** 10.1098/rstb.2022.0335

**Published:** 2023-09-25

**Authors:** Adam Zaidel, Roy Salomon

**Affiliations:** ^1^ Gonda Multidisciplinary Brain Research Center, Bar-Ilan University, Ramat Gan 5290002, Israel; ^2^ Department of Cognitive Sciences, University of Haifa, Mount Carmel, Haifa 3498838, Israel

**Keywords:** Bayesian, perception, interoception, exteroception, body

## Abstract

Classic Bayesian models of perceptual inference describe how an ideal observer would integrate ‘unisensory’ measurements (multisensory integration) and attribute sensory signals to their origin(s) (causal inference). However, in the brain, sensory signals are always received in the context of a multisensory bodily state—namely, in combination with other senses. Moreover, sensory signals from both interoceptive sensing of one's own body and exteroceptive sensing of the world are highly interdependent and never occur in isolation. Thus, the observer must fundamentally determine whether each sensory observation is from an external (versus internal, self-generated) source to even be considered for integration. Critically, solving this primary causal inference problem requires knowledge of multisensory and sensorimotor dependencies. Thus, multisensory processing is needed to separate sensory signals. These multisensory processes enable us to simultaneously form a sense of self and form distinct perceptual decisions about the external world. In this opinion paper, we review and discuss the similarities and distinctions between multisensory decisions underlying the sense of self and those directed at acquiring information about the world. We call attention to the fact that heterogeneous multisensory processes take place all along the neural hierarchy (even in forming ‘unisensory’ observations) and argue that more integration of these aspects, in theory and experiment, is required to obtain a more comprehensive understanding of multisensory brain function.

This article is part of the theme issue ‘Decision and control processes in multisensory perception’.

## 1. More than multisensory

Imagine yourself sitting on the beach. Despite the busy scene, with some people playing beach-bats nearby, you discern the sound of a chirp and spot a green parrot sitting on a branch. Classical theories of multisensory processing inspect such cases and define the rules for integration (or segregation) of the audio-visual signals [1,2]. However, the full inference process is much more complex than typically assumed. We often take for granted a multitude of processes underlying our ability to sense (and make sense) of the external world. For example, for stable vision, the brain uses efferent information to cancel out bodily and ocular motion [3–6]. Moreover, internal sensory information conveyed from proprioception (e.g. where are my eyes looking?), vestibular (e.g. how is my head positioned?) and interoception (e.g. the sound is external and not my heartbeat) is seamlessly and implicitly taken into account to allow the explicit sensory experience of the world around us [7–9]. All our sensory experiences are in actuality based on both internal and external multisensory information.

## 2. The fallacy of ‘unisensory’ perception

Perception of objects and events in the environment is a vital skill for survival and everyday behaviour. To achieve this, the brain needs to perform a multitude of perceptual decisions, on an ongoing basis [10–13]. In doing so, it must filter the constant barrage of signals from our various senses and interpret them appropriately. A solid theoretical foundation, based on signal detection theory and Bayesian statistics, has underscored major advancement in our understanding of perceptual decision making in the brain in the past few decades [14–21]. The rationale behind this computational approach is that signals received from our senses are noisy and to interpret them the brain needs to solve a statistical optimization problem—specifically, to infer their most likely cause(s).

Armed with these powerful analytical tools, classic psychophysics experiments have been interpreted in terms of, and used to quantify, the brain's ability to measure a plethora of sensory and perceptual features [22–26]. Furthermore, Bayesian theories of perceptual inference explain how the brain integrates separate measurements from the individual sensory cues into a unified percept (multisensory integration) [27–33]. For experimental control, other factors, besides the feature(s) being measured, are typically held constant in laboratory conditions (e.g. eye fixation, synthetic stimuli with custom manipulations). Such measurements are often loosely termed ‘unisensory’ because they measure responses to stimuli experimentally controlled in one specific modality. However, it is imperative to note that our other senses can never be turned off.

Even when one's eyes are fixated, and the head is stationary, proprioceptive signals from the eye muscles and vestibular signals regarding head position continue to bombard the brain [8,9]. In complete darkness, the visual cortex remains highly active (and this activity even differs with eyes open or closed [34]). Interoceptive and tactile signals are always present [7]. Thus, ‘unisensory’ perception is a misnomer. In ‘unisensory’ experiments (or measurements), signals from the sense being probed are always received in combination with signals from the other senses and interpreted within that multisensory context. Thus, while we may decide to manipulate sensory signals and ask for decisions in one sensory domain in our experiments, we are always in practice impacting multiple sensory streams. Put simply, there is no, nor has there ever been, true unisensory stimulation.

## 3. A shortfall of the current Bayesian multisensory perspective

The Bayesian framework of perceptual inference has been incredibly valuable and influential for understanding multisensory integration. However, there is a gap between the way it is modelled and tested in the laboratory, and actual multisensory brain function. First, Bayesian multisensory integration models rely on receiving two (or more) ‘unisensory’ cues that undergo integration. But, as described above, there are no pure ‘unisensory’ cues in the brain. Rather, each unisensory cue exists within a multisensory context and therefore is itself an outcome of multisensory processing. This creates a problem of circularity.

**Figure 1.**
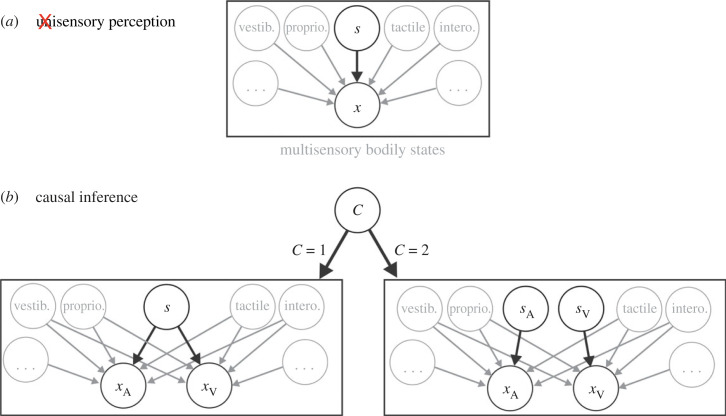
Perception is inherently multisensory. (*a*) Black components present the simplest generative Bayesian model (‘unisensory’ condition). Parameters *s* and *x* represent the stimulus and observation, respectively. The stimulus (*s*) can be described by the probability distribution *p*(s). The observation is dependent on the stimulus (black arrow) and has a probability distribution *p*(x|s) reflecting sensory noise. The grey circles and arrows depict other modalities that influence the ‘unisensory’ observation, which are typically ignored. (*b*) A Bayesian causal inference model to determine whether an auditory and a visual measurement (*x*_A_ and *x*_V_, respectively) were caused by one source (*C* = 1) or two separate sources (*C* = 2). Here too, the measurements are each influenced by other modalities (grey circles and arrows) typically not taken into account. (Online version in colour.)

Let us consider this from the perspective of building a Bayesian model of perceptual inference. The first step is to specify the ‘generative model’ [35] (figure 1)—a statistical description of how the sensory observations come about. In its simplest form, this comprises a stimulus (*s*) and the brain's measurement thereof (*x*, an ‘observation’). This represents the simplest ‘unisensory’ case (the black components in figure 1*a*). The arrow leading from *s* to *x* indicates that the observation depends on the stimulus. But, in reality, the observation also depends on all the other sensory systems (grey circles and arrows in figure 1). Bayesian theory has a way to deal with these ‘nuisance’ parameters (via a process called marginalization [36]). However, dimensionality of the problem increases with the number of parameters, and their values are usually unknown. Even just identifying all the influential parameters is intractable. So, in practice, these multisensory dependencies are largely ignored.

Beyond the practical problem of needing to marginalize over all the senses and states, there is also a conceptual problem in that Bayesian multisensory models purport that some brain circuit receives ‘unisensory’ observations as input. For example, the well-known ‘causal inference’ model [2] (figure 1*b*) describes how best to decide whether two unisensory observations (e.g. audio, *x*_A_ and visual, *x*_V_) were caused by one source (*C* = 1, bottom left panel), in which case they should be integrated, or two separate sources (*C* = 2, bottom right panel), in which case they should not be integrated. But these ‘unisensory’ observations are in fact each a result of multisensory processing. They depend on the states and input from other senses (grey circles and arrows in figure 1*b*), e.g. proprioceptive signals from the eyes and neck, vestibular signals from the head, and corollary discharge from intended movements, all strongly influence visual and auditory observations [5,6,37–41].

Moreover, sensory systems are not passive receivers. Rather, they are part of sensorimotor loops. Not only do the movements we make influence sensations, humans and animals actively move to sense the environment (active sensing) [42,43]. We move our eyes to probe the visual scene and move our hands to generate tactile input. This means that sensory signals can only be interpreted within a sensorimotor context, in addition to the mixing of signals from multiple senses. Thus, the observer must fundamentally determine whether each sensory observation is from an external (versus internal, self-generated) source to even be considered for integration. This creates a chicken-and-egg problem, in which multisensory integration and causal inference are needed to supply the inputs for multisensory integration and causal inference.

## 4. Mixed signals from the start

Mixing of multisensory signals begins very early in the neuronal hierarchy. Anatomically and physiologically, multisensory signals reach far into the sensory periphery. Even lower-level reflexes are altered by multisensory signals. The pupillary light reflex is larger for multisensory stimuli [44–46]. Thus, multisensory signals affect visual inputs even before they reach the retina. In the auditory and vestibular systems, efferent signals from the brain reach all the way to the sensory end organs [47–49]. Hence, even signals from the sensory end organs are not strictly ‘unisensory’.

Seminal work on multisensory integration in the superior colliculus exposed subcortical convergence of visual, auditory and somatosensory signals in single neurons [50]. Interestingly, some of these features are not present at birth, but rather develop with experience in a multisensory world [51]. However, not all multisensory function is learned. Certain aspects are innate and present irrespective of experience [51,52]. Also, some aspects, such as ‘superadditivity’ (when the response to the combined cues exceeds the sum of the responses to the single cues [53]), seem to be mainly a feature of subcortical multisensory processing, and less cortical [54].

Cortically, multisensory signals are seen already in early (once considered ‘unisensory’) areas [55–58]. These areas even have cross-modal interconnections [59–61]. As one moves up the cortical hierarchy, multisensory responses in single neurons become ubiquitous. Notably, however, the types of interactions are heterogeneous across (and even within) areas. For example, neuronal responses to visual and vestibular stimuli in extrastriate visual area MSTd (medial superior temporal dorsal part) reflect summation of signals in some (congruently tuned) neurons, but diminished sensitivity in other (oppositely tuned) neurons [62]. In the visual posterior Sylvian area, visual and vestibular responses in single neurons more commonly have opposite tuning [63]. In the posterior parietal area, ventral intraparietal heterogeneous visual and vestibular responses are seen, with strong decision-related activity [64–66]. Lastly, cross-modal (visual–vestibular) recalibration reveals very different phenomena across different multisensory areas [67,68]. During the same perceptual recalibration, ‘unisensory’ tuning (to the same cue) can shift in opposite directions in two different brain areas [68].

## 5. Multiple stages of heterogeneous multisensory processing

Multisensory processing therefore occurs all along the brain hierarchy, where each stage (and modality) requires specific and heterogeneous functions. Although ‘multisensory integration’ is often used as an umbrella term, we think that it should be reserved for the specific case in which two (or more) underlying cues' measurements of a stimulus are combined into a unified estimate [33]. For a general term, we prefer ‘multisensory processing’ (see table 1 for a list of common multisensory terms, and their meanings, as we use them here). Take for example a task of estimating the location in space of a brief auditory stimulus. An observer will need to take into account their current head position at the time of the stimulus. Hence, it is a multisensory process. But here, proprioceptive signals from the neck and body do not carry information regarding the event *per se*. Thus, they are not integrated together with the auditory measurement. Rather, the auditory measurement is interpreted within their context, to obtain an allocentric location estimate. Hence, we would not consider this ‘multisensory integration’ in the strict sense of the term (rather, this is a different type of multisensory processing).

**Table 1.** Multisensory terms.

term	meaning
multisensory processing	a host of brain functions that deal with multiple sensory modalities' inputs and states—for a variety of different purposes, including integration, suppression, etc.
multisensory integration	combining multiple sensory measurements from different modalities into a unified estimate of the stimulus
multisensory suppression	the neuronal or behavioural responses to a stimulus are reduced by the presence of another stimulus from a different modality
multisensory interactions (or dependencies)	when the response or measurement from one modality depends on the state (or input) of another
multisensory causal inference	probabilistic inference regarding the origin(s) of the experienced stimuli
multisensory recalibration	shifting the estimates of one or more sensory modalities in relation to each other or to the environment

The classic case of multisensory causal inference (e.g. to determine whether or not an external visual and an auditory observation reflect the same source; figure 1*b*) lies toward the upper end of this computational hierarchy. Its inputs have presumably already undergone other basic multisensory processing to determine that they are both of external (versus internal) origin—e.g. the chirp and the parrot from the example at the opening of this article. If the sound resulted from one's own action, then it would be irrelevant to consider whether it came from the same source as the visual observation. Thus, fundamental multisensory processes need to take place before higher-level causal inference and/or multisensory integration are invoked (or at least be a part of these processes). This notion, that the brain's signals already from the periphery can only be understood within a multisensory sensorimotor context, amplifies many questions regarding multisensory processing. How does the brain ‘know’ at each stage how to handle these signals? How do these functions develop in the absence of pure unisensory signals? Does the process to determine whether stimuli are of external versus internal origin need to happen before causal inference (to attribute the stimuli to one or more sources) can be invoked? Or can these inferences happen in parallel?

## 6. A primary mission to dissociate self- versus externally generated signals

Perhaps the most fundamental distinction regarding sensory signals is whether these are internally generated by the organism itself or arise from the environment [69]. This inference is often taken for granted under most multisensory processing schemas, yet the process of delineation of the sensory signals from the organism or the outside world is computationally complex. One must remember that these sensory streams are constantly entwined and there is no known *a priori* labelling of internal versus external signals, but rather these must be learned and inferred from experience.

**Figure 2.**
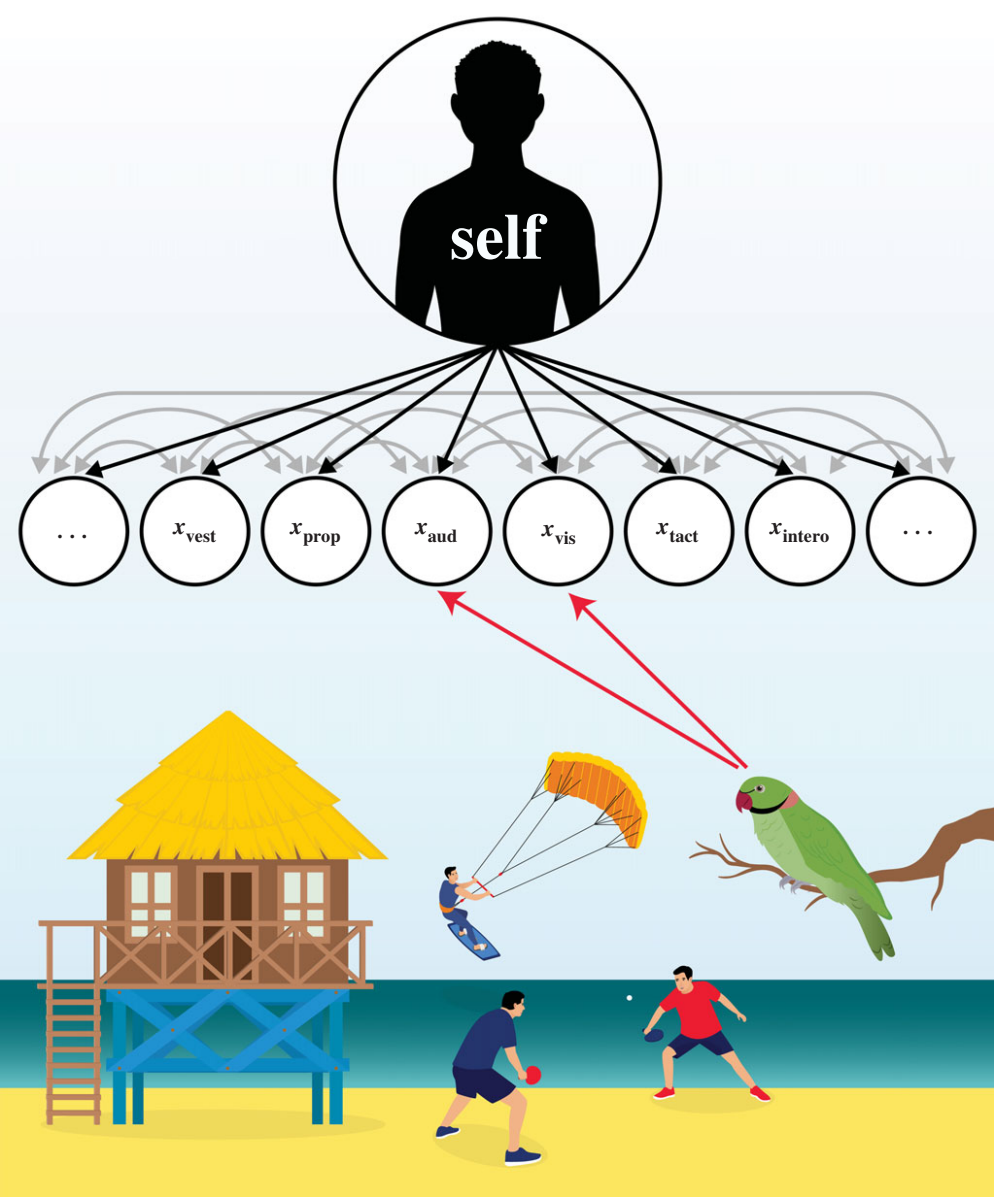
Observations are driven by internally and externally generated signals. Sensory measurements (black circles) are dependent on internally generated signals (black arrows), externally generated signals (e.g., originating from the parrot; red arrows) and cross-modal influences (grey arrows).

At any given moment, our sensory measurements concurrently reflect a mixture of internally generated signals, externally generated signals and cross-modal influences (black, red and grey arrows, respectively, in figure 2). Thus, returning to the beach example (from the opening of this article), fundamental to performing causal inference for the external signals (whether or not the visual and auditory observations both came from the parrot, figure 2), one must determine that these signals indeed come from the environment. How then, can this primary case of causal inference, enabling the delineation of the self and the world, come to be?

## 7. Multisensory interactions bound in the bodily self

William James famously noted that our body, ‘is always there’ [70], denoting the primacy of the bodily self in experience. The basic embodied sense of selfhood is considered pre-reflexive (i.e. it does not require explicit awareness or attentional resources) and phenomenologically transparent [71,72]. Current accounts suggest that predictive processing of multisensory signals may underlie the formation of coherent bodily self models [73–75]. Such accounts thus propose that the self is formed through predictive models of the integration of exteroceptive [76–78], volitional [79–82] and interoceptive signals [83–87].

Empirical work has revealed that the bodily self is achieved and maintained via complex interactions among interoceptive and exteroceptive signals [76,88–91]. For example, body ownership, the experience of being in a physical body [71], has been shown to rely on interactions between multisensory signals. Experimentally, illusory body ownership can be induced when viewing touch on a rubber or virtual limb that is coupled with synchronous touch on one's unseen real limb [91,92]. In such cases, the brain is exposed to conflicting multisensory information (e.g. the rubber hand is not in the location of one's real hand) but the visual and tactile signals are synchronous, and the brain must resolve this conflict by making a causal inference regarding the most probable origins of the signals. Interestingly, in this case, the conflict is typically resolved in favour of the corresponding visual–tactile signals and the rubber hand is felt as belonging to the self, demonstrated by behavioural, physiological and brain responses [90,92–95]. Similarly, illusory ownership may be induced by cardio-visual synchrony [95,96]. Indeed, recent accounts have suggested that body ownership may be accounted for using causal inference principles [95,97–99]. According to this approach, based on rubber hand illusion experiments, body ownership is computed using a causal inference process, where if a common cause is inferred for visual, tactile and proprioceptive signals, the rubber hand is perceived as part of the self [97,98]. However, if the visuo-tactile stimulation is asynchronous, the rubber hand is rotated or distanced, this illusion dissipates. Thus, the brain combines prior expectations regarding the multisensory statistical probabilities with incoming sensory signals to decide if these should be bound together as occurrences happening to the ‘self’.

## 8. Sensorimotor expectations

Similarly, self-generated, volitional actions provide robust signals for segregation of the self from the environment [3,69,100]. Predictive accounts based on the ‘comparator model’ suggest that volitional actions produce forward models which in turn predict the afferent sensory signals arising from that action [101,102]. The predicted and afferent sensory signals are then compared and, if they match, the sensory effects can be attributed to the self. These are then often cancelled out at both the perceptual [103–105] and neural level ([106,107], but see [108]). A classic example of such predictive causal inference, first noted by von Helmholtz [3], is the cancellation of the effects of self-generated oculomotor and bodily motions on visual information [3,4]. While we make constant movements with our eyes, head and trunk, the resulting movement on the retinal surface is attenuated, affording us a stable perception of the world. Similar predictive mechanisms are thought to enable the segregation of exafferent (world-generated) versus reafferent (self-generated) sensory signals across numerous modalities. In humans, the sense of agency, our feeling of control over our actions, is similarly thought to arise from predictive processing of sensorimotor signals [80,92,93,109], allowing explicit segregation of the self from the world through volitional actions [72,110–113].

## 9. Interoceptive signals

In recent years, research regarding the impact of interoceptive information such as cardiac, respiratory and visceral signals on perception [84,114,115] and the construction of self models [83,85,89,116,117] has flourished. At the theoretical level, it has been proposed that predictive processing of interoceptive signals, critical for homeostasis, may serve as the basic scaffold for the embodied self [86,89,118–120]. Indeed, under normal conditions, interoceptive signals are supressed from explicit awareness [84,121,122] and have been shown to be modulated by experimentally induced changes in the bodily self [83,88,123]. This suggests that they are a fundamental aspect of the self model. In fact, some researchers have proposed that interoceptive signals driving homeostatic regulation may have a privileged status for maintaining an organism's stability and as such may serve as ‘first priors’ (fundamental rules guiding learning) [86]. It is likely that these signals undergo similar causal inference processes early in development, enabling the precise predictions required for homeostatic survival. Moreover, recent evidence has shown that active sampling of the external world by exteroceptive senses such as vision and touch are unconsciously timed to the cardiac cycle to allow better signal-to-noise differentiation [124,125].

Taken together, these perspectives point to predictive inference processes, integrating rich multisensory information, as the driving impetus for the formation of a sense of bodily self. As such, the self can be viewed as a spatio-temporal construct for which the brain has developed predictive capacities regarding sensory signals, based on learned models of their co-dependencies. This fundamental model can later serve to make the primary distinction between the self and the world, allowing more fine-grained inferences regarding the origins of sensory signals. But how are such models acquired?

## 10. Development of a self model

While the precise processes underlying the acquisition of a multisensory model of the self remain unknown, we can denote several types of mechanisms by which this is formed. Many aspects of multisensory interactions and integration are biologically hard-wired into the neural system. For example, Gibson and others have proposed that the infant brain is inherently multisensory [126,127]. Neuroanatomical structures, such as the superior colliculus, have converging inputs from visual, auditory and tactile modalities, allowing multisensory interactions at early stages of neural processing [50,128]. Similarly, reflexes, present in early postnatal development, attest to an innate link between different sensorimotor modalities [129]. Moreover, reflexes have been shown to be modulated by self in addition to external stimulation [130]. Despite these innate aspects, it is clear that building a proficient model of multisensory dependencies is experience-dependent [51,128], with increased refinement during development. Moreover, there is evidence that learning of a multisensory model underlying the self may even begin *in utero* [87,131,132].

This multisensory experience, however, is not passive but is driven by both instinctive and volitional actions causing sensory signals. Volitional actions, in particular, hold special potential for construction of the self model as they allow high-precision inference regarding the predicted consequences of actions. Indeed, many predictive coding accounts suggest that volitional actions have a central role in the acquisition of a generative model of the self [111,132–135], by allowing directed exploration of co-dependencies between sensory signals [136,137]. Thus, the bodily self may serve as a ‘first prior’ binding together multisensory signals and allowing a distinction between self-generated signals and those arising from the external world. Moreover, the organism needs to dynamically maintain (recalibrate) its multiple sensory systems on an ongoing basis [138–140]. Once these fundamental strata of (internal) multisensory processing are accomplished, the senses can be directed to extract information about the (external) world.

## 11. Loops of multisensory processing

The formation of an implicit self model, based on inferences from the acquired sensorimotor predictions, may then enable further forms of multisensory inference. First, learned multisensory dependencies may allow marginalization and differentiation of ‘unisensory’ signals. For example, once retinal movement related to self-generated motion is accounted for and intrinsically modelled, one may attribute other forms of retinal movement as relating to movement in the external world. Across all sensory modalities and interactions, the subtraction of predicted sensory signals arising from intrinsic correspondences and actions (self model) allows distillation of specific sensory streams. Over time, this process could allow the differentiation of ‘unisensory’ signals for perceiving the external world. Indeed, our experience of bodily sensations (e.g. proprioception, interoception and vestibular) are typically not the focus of our explicit awareness [141–144], which is directed toward the external world. Correspondingly, this transparent phenomenology causes semantic representations of these internal, self-related signals to be severely impoverished in comparison with exteroceptive sensory signals. Thus, in any given moment (figure 2), our explicit awareness will typically be focused on an external object in the world (I see a parrot) while the multitude of sensory interactions enabling this sensation are bound under an implicit model simply denoted as 'I'. The attenuation of the self model is consistently found in both phenomenology and the brain [84,104,106,107,145], allowing enhanced perception of external stimuli.

## 12. Future outlook

In summary, the common assumption that the brain receives unisensory signals from the environment is false. Accordingly, the questions of causal inference and multisensory integration of these signals are only the ‘tip of the iceberg’. Below, supporting these functions, lies a complex set of inherently multisensory processes. First, the organism needs to form a model of self, to be able to fundamentally determine which stimuli are externally versus internally generated. This model is acquired through learned multisensory and sensorimotor dependencies, and built on a neural substrate with the innate potential to make these connections. Then, only after early multisensory processing to determine external (versus internal) origins, to distil the signal from one's own actions, and to marginalize over other sensory states, do classic causal inference and multisensory integration of signals from the environment become relevant.

Some experiments and modelling may still need to assume unisensory inputs. And of course, such studies have been paramount to building our foundational understanding of multisensory processing. Thus, our point is not to criticize this practice. Rather, we aim to highlight the weight (and limitations) of the assumptions. And, to call attention to the multisensory processes that take place all along the neural hierarchy, often ignored in modern multisensory modelling. Multisensory processing is not just a high-level function. It is fundamental to being. Thus, the scientific questions regarding multisensory brain function have perhaps been largely understated, and their scope underestimated.

Greater appreciation of this might open up new avenues for research. First, by connecting between researchers across different disciplines, this will force the refinement of semantics (sometimes the same term has different meanings, or the same phenomenology or computation has different terms) and the broadening of horizons to the full scope of the multisensory problem. It may also help elucidate open questions in the field, such as ‘opposite’ (versus ‘congruent’) multisensory tuning, and contrary neuronal recalibration in different brain areas [68]. Internal models built on our own bodily experience are also used to perceive other people in the world [146,147]. Thus, how causal inference to determine internal versus external stimulus origin is used or influenced by this function is an open question. Lastly, many disorders of brain function have been linked to aberrant ‘unisensory’ or multisensory function or causal inference, e.g. autism [148–151], Parkinson's disease [152,153], schizophrenia [109,154] and many more. One therefore needs to be cognizant of the fact that all ‘unisensory’ performance includes a measure of multisensory processing, and that testing a specific multisensory function is just one aspect amongst a broad range of heterogeneous functions. Better integration (on our behalf) of these disparate aspects of multisensory processing is essential for a comprehensive understanding of the brain, in health and disease.

## Data Availability

This article has no additional data.
